# Student Expectations and Outcomes in Virtual vs. In-Person Interprofessional Simulations: A Qualitative Analysis

**DOI:** 10.3390/nursrep15030114

**Published:** 2025-03-20

**Authors:** Padmavathy Ramaswamy, Abbey M. Bachmann, Tiffany Champagne-Langabeer, Chasisty L. Gilder, Samuel E. Neher, Jennifer L. Swails

**Affiliations:** 1 Department of Graduate Studies, Cizik School of Nursing, UTHealth Houston, Houston, TX 77030, USA; 2 Department of Educational Programs, McGovern Medical School, UTHealth Houston, Houston, TX 77030, USA; abbey.m.bachmann@uth.tmc.edu (A.M.B.); samuel.e.neher@uth.tmc.edu (S.E.N.); 3 McWilliams School of Biomedical Informatics, UTHealth Houston, Houston, TX 77030, USA; tiffany.champagne@uth.tmc.edu; 4 Tilman J. Fertitta Family College of Medicine, University of Houston, Houston, TX 77204, USA; cgilder@central.uh.edu; 5 Department of Medicine, Emory University School of Medicine, Atlanta, GA 30303, USA; jennifer.lee.swails@emory.edu

**Keywords:** interprofessional education, teamwork, simulation, qualitative, virtual simulation, nursing

## Abstract

**Background**: Health-related programs frequently integrate interprofessional education (IPE) into their training. The COVID-19 pandemic transitioned many IPE programs online, making it essential to assess student expectations and perceived learning outcomes across virtual simulations and in-person settings. **Methods:** This qualitative study compared student expectations and self-reported outcomes across in-person and virtual case scenarios at a Texas health science center. Responses to open-ended questions from two data collection periods were analyzed using inductive coding and thematic analysis. **Results:** Students from nursing, medicine, dentistry, public health, and informatics participated in each group. Three major themes emerged from this study: communication, teamwork, and role identification, with self-development and professionalism as major subthemes. For communication, students often described a desire for increased simulations to “practice with interprofessional communication”. Teamwork was the second theme identified, with students discussing the significance of effective teamwork, such as, “It is a good practice to work together, listen to each other, and achieve a common goal of patients getting better”. Additionally, students expressed a desire to better understand the roles of other healthcare professionals across different settings. **Conclusions:** Realistic IPE simulations may help students build confidence in their team roles while understanding other health professions. To strengthen curriculum design, faculty should include student expectations and perceived outcomes from IPE activities. A limitation of this study is the reliance on self-reported data, which may introduce response bias and the potential variability in student experiences.

## 1. Introduction

Interprofessional education (IPE) occurs when a team of learners from diverse fields work together to learn and solve a problem [1]. Constructivism learning theory explains that learning occurs through building on prior knowledge and from social interactions with others [2]. Additionally, according to Kolb’s experiential learning theory, learners’ knowledge is created by the process of experience transformation, consisting of four stages: concrete experience, reflective observation, abstract conceptualization, and active experimentation [3]. Experiential learning theory has been found to be an effective theory for understanding how learning occurs in IPE [4]. At UTHealth Houston, Texas, USA, through the Center for Interprofessional Collaboration (CIPC), students from six schools within the university have interprofessional activities embedded in their curriculum. Many interprofessional activities through the CIPC moved online during the COVID-19 pandemic. Two activities completed in person before the pandemic and virtually during the pandemic included a case of medical error in an inpatient setting. Another case focused on an uninsured patient seen in an outpatient clinic. As in-person learning opportunities became possible again, deciding which modality to use became a question, and more research was needed to support one delivery method over the other regarding student expectations and outcomes. In both cases and both settings, students completed a pre- and post-survey. The researchers used student responses from these questions to examine the following central question: Are the expectations of interprofessional education activities different when students attend in person compared to virtually? The following research questions guided this study:

1. What do students expect to learn when completing an in-person IPE activity compared to completing a virtual IPE activity?
2. How do the self-reported outcomes of students who completed an in-person IPE activity differ from those who completed a virtual IPE activity?

This study also explored and compared the student expectations and learning outcomes of two distinct case scenarios—an outpatient charity care and an inpatient medication error scenario.

### Background

The World Health Organization (WHO) Framework for Action on Interprofessional Education and Collaborative Practice [5] states that “Interprofessional education occurs when two or more professionals learn about, from, and with each other to enable effective collaboration and improve health outcomes” (p. 7). Learning alongside peers from other professions in collaborative teams creates space for students to gain insights into the contributions of different team members and work together more effectively, as they would in their future healthcare settings. One way that learners can practice IPE is through engaging in simulation activities. Research suggests that the sooner IPE is embedded into the student’s learning experience, the more impactful it can be on the student’s perceptions of their role in healthcare [6]. While there is a significant amount of research to support the implementation and impact of IPE on learning experiences, more research is needed on student feedback and self-reported outcomes comparing the in-person and virtual modes before and after the COVID-19 pandemic.

Prior to the global impact of COVID-19, IPE was a developing phenomenon in healthcare education. In-person IPE simulations posed challenges such as ensuring that faculty were accurately trained, creating meaningful activities, resources, curriculum, and accreditation requirements, and cross-collaborating multiple schools and disciplines [7]. COVID-19 added another layer of challenge when all learning experiences were expected to transition seamlessly into virtual formats. Recent research has focused on learned and lived experiences and the global pandemic’s effects on IPE. McKinlay et al. discussed the strategies and skills needed to keep IPE simulations going during such a time. These included foreseeing potential challenges, staying committed to continuing IPE throughout the pandemic, ensuring student support, using technology appropriately, and measuring outcomes [8]. Power et al. (2022) described learned and lived experiences from transitioning to a virtual environment and provided tips on delivering IPE simulations. These included guidance, support, clear instructions, interaction and supervision, and considering the student’s feedback [9]. The transition process for many institutions was not all bad. Some found that the virtual format solved prior issues, such as finding a venue for a large group of participants and travel time for students and facilitators. A virtual setting provided an optimal venue for convening large groups while continuing to deliver authentic IPE [10].

The current study compared in-person and virtual formats of two interprofessional simulations, shedding light on how delivery format impacts student expectations and outcomes. Qualitative student feedback was carefully examined via post-simulation feedback surveys from in-person and virtual cases to investigate the two interprofessional simulation cases. A previous study analyzed the quantitative data using the same two case simulations [11]. One case focused on an inpatient scenario with a medical error, and the other focused on a patient scenario in a charity clinic. Both cases required student teams to huddle during the simulation. The charity case required the team to make a rapid decision to send a geriatric patient to the emergency room, and the other case required the student teams to break the news of a medical error to a seemingly outraged patient. This study found that in the virtual setting, the medical error case study maintained a large effect size (0.81). At the same time, the charity care simulation had a lesser impact with a smaller effect size (0.64) [11]. The consequences of transitioning from in-person to virtual simulations from the student’s perspective are critical to deciding whether modifications are needed in the future. Students provided detailed feedback after participating in two different IPE simulations, one in-person and one virtual simulation. IPE learning experiences encourage better communication and collaboration in the future careers of healthcare students and are essential for quality care. Qualitative feedback results can demonstrate student learners’ engagement [12]. This study was conducted to satisfy that need. This study examines the qualitative feedback from the same two case simulations and offers insight into the expectations and self-reported outcomes of student learners completing in-person and virtual simulations.

## 2. Methods and Materials

The UTHealth Houston, Texas, USA Institutional Review Board approved this study as quality improvement (HSC-MS-19-0362). Informed consent was obtained from all participants involved in the study. Students were informed that their participation in the study was embedded in the core curriculum as a quality improvement initiative to improve educational practices. The purpose of the study was clearly explained, as were the objectives. Students were informed that their grade was based solely on participation and not on their responses or performance. They were told explicitly they could stop at any time without penalty or impact on their grade and that personal data would be deidentified. This study used two case scenarios completed by students from across the university; each was completed in person and virtually. The research team analyzed qualitative data from the pre-/post-test surveys.

### 2.1. Case Scenarios

A team of interprofessional faculty created two case-based scenarios. The process of creation and validation of the case scenarios has been published elsewhere [13]. Participants and faculty were from various schools, including medical, nursing, dentistry, public health, and health informatics. One case-based scenario focused on a medical error in an inpatient setting, and one focused on an uninsured patient seen in an outpatient charity clinic. The in-person simulations occurred in the academic year of 2019–2020 and the virtual ones were in the academic year of 2020–2021. The goals of these case-based simulations included improved collaboration, teamwork, communication, and a greater understanding of the roles of other healthcare providers.

The outpatient case scenario begins with students engaging in a team huddle regarding an elderly patient scheduled to be seen in a charity clinic. The patient recently lost her health insurance and complains of tooth pain. Upon entering the room, the students learn of a new complaint of back pain and receive results from radiology that the patient has a vertebral fracture and a possibility of severe spinal cord injury. Students must quickly change their initial plan and make a quick decision regarding the immediate transfer of the patient to the emergency room. The inpatient medical error case scenario starts with students performing a root cause analysis before entering a patient room to disclose a medication overdose to the patient and a family member. The patient, who is under the impression that they are being discharged, reacts significantly upon hearing the change in status.

In both case scenarios, students were given information about the patient, including name, setting, chief complaint, and vital signs (Supplementary S2 and S3). Standardized patients (SP) received training from the simulation lab staff and faculty to depict realistic patient and family interactions and display intense emotional responses during these encounters. They debriefed with students after the simulation, providing insight and feedback for handling similar situations in their future practice. In both cases, students used team skills to form a shared mental model, support each other, and collaborate to respond to the evolving situation. The complete simulation exercise included the case scenarios, an SP debrief, a faculty debrief, and time for reflection [13].

### 2.2. Participants

The students were from various schools at UTHealth Houston, Texas, USA. Student participation in these simulations was mandatory for fourth-year medical students, undergraduate nursing students (inpatient scenario), graduate nursing students (outpatient scenario), and third-year dental students, and optional for students from the schools of public health and clinical informatics.

The in-person outpatient charity case had a sample size of 488 students in 2019–2020, while the sample size for the virtual administration of this case in 2020–2021 consisted of 528 students. A sample size of 520 students was reflected in 2019–2020 as part of the in-person medical error simulation as compared to the 236 students who completed the virtual medical error simulation in 2020–2021.

### 2.3. Data Collection

Qualitative data were collected using open-ended questions incorporated in the pre-test and post-test surveys administered before and after the simulation (Supplementary S1). Questions in the pre-test survey pertained to what students hoped to gain from the simulation experience. The open-ended questions in the post-test survey pertained to what students learned from the activity. Before each simulation, students were asked, “What are you hoping to gain from this experience?” After completing the case simulations, students were asked, “What did you learn from this IPE activity that you can apply to your professional career?” Students were enrolled in an online course through a learning management system (LMS) for both data collection periods. The first collection period was from August 2019 to May 2020, when the simulations were held in person. The second collection period was from August 2020 to May 2021, when the simulations were transitioned to virtual, synchronous video conferences [11].

### 2.4. Data Analysis

The responses to the open-ended questions from the two data collection periods (2019–2020 and 2020–2021) were downloaded to Excel by the simulation coordinators. Two authors (AB and SN) analyzed data from the inpatient case scenario. SN analyzed the data from the 2019–2020 data collection period when the simulation was held in person, and AB analyzed data from 2020 to 2021 when the simulation was conducted virtually. Similarly, two authors (CG and PR) analyzed data from the outpatient case scenario; PR analyzed data from 2019 to 2020 (in-person), and CG from 2020 to 2021 (virtual). All of the authors are experienced in conducting qualitative research.

The authors used inductive coding, which involves creating codes based on the data [14,15]. Each author engaged in line-by-line coding for each participant’s response to each open-ended question to identify codes representing patterns of meaning in the data [14,15]. These codes were developed through repeated data analysis; responses were assigned a number 1 if they pertained to a specific code, establishing new codes for each response that did not fit into a previously established code [16]. These codes were then cross-reviewed by another member of the research team. Any discrepancies noted in coding were color-coded for review by the entire research team. The research team met to review and resolve coding discrepancies before discussing how the codes for each set of data (the in-person and virtual inpatient case scenario and the in-person and virtual outpatient case scenario) aligned. Any new codes developed in this process included an additional review by the initial coder to ensure all data aligned with the additional codes.

A final review by the research team took place to determine any remaining overlap in codes across case scenarios and virtual and in-person approaches. All labeled codes were compared across all four separate codes to determine overlap. Codes determined to be in the same category were collapsed into each other and given an all-encompassing title. For example, three of the four research team members had created a “teamwork” code, while a third researcher had a code labeled “collaboration”. After discussing the intention of the code labels with the team, “collaboration” was determined to have the same meaning as “teamwork” and was therefore recorded as “teamwork”. This approach allowed the research team to ensure continuity and consistency in codes across case scenarios and members of the research team before conducting a count of the frequency of codes to determine categories and accompanying themes.

While this methodological approach allowed for student perspectives to be shared openly via self-reported data, we acknowledge that this also presents a challenge in that the data may include biased responses and provides a wide variability in student responses.

## 3. Results

For the outpatient charity care simulation conducted in person in 2019–2020, 488 participants completed the pre-test survey, while 485 (99%) participants answered the post-test survey. For the same outpatient simulation in a virtual setting during 2020–2021, 528 students responded to the pre-test survey, and 516 (98%) answered the post-test survey. The inpatient medical error simulation completed in person in 2019–2020 garnered 520 responses as part of the pre-test and 492 (95%) responses for the post-test survey. In the virtual inpatient simulation in 2020–2021, 236 pre-test responses were received compared with 229 (97%) post-test survey responses. The majority of students completed both the pre-test and post-test surveys, providing a high survey response rate. Demographics of students participating in the simulations for the two data collection periods are described elsewhere [11]. Before each simulation, students were asked, “What are you hoping to gain from this experience?” After completing the case simulations, students were asked, “What did you learn from this IPE activity that you can apply to your professional career?” After qualitative data responses were analyzed, student expectations for both simulation cases on their pre-simulation survey were revealed and compared to student perceptions of the outcomes of both simulation cases in their post-simulation survey. Three main themes emerged from the data analysis: communication, teamwork, and role identification. The theme of communication was captured by 18.1% (n = 312/1722) of all student participants. The theme of teamwork appeared in 62.3% (n = 1072/1722) of total student responses, while exclusively appearing in the self-reported outcomes for 55.8% (n = 959/1722) of students. Role identification was a key theme for the outpatient case scenario, captured by 15.1% (n = 151/997) of students in the pre-test and 20.1% (n = 201/999) of post-test student responses. The themes and subthemes are described below.

### 3.1. Theme 1: Communication

Students agreed (n = 312/1722; 18.1%) that they hoped to receive better insight into the best communication approaches as they participated in both the virtual and in-person simulation cases. They often described their hopes for the simulations: [to] “*practice with interprofessional communication*” and extend their current “*abilities to collectively collaborate*” with the healthcare professionals in their respective interprofessional simulation groups. Some students even extended their expectations with the goal of using their communication skills to “*facilitate patient care through flawless multidisciplinary communication*” and expressed the hope of “*developing more interprofessional and patient communication skills and experience*”. Students continually echoed the sentiment that they hoped to use the improved communication skills “*in future teams*”.

In alignment with what students expected to gain from the case simulations in both virtual and in-person approaches, communication was a typical response (n = 716/1722 41.6%) in some of the best practices students learned after working with an interprofessional team in a simulated clinical experience. Some students mentioned the importance of communication within teams and with patients, as evidenced in the response, “*I learned the importance of communication within a team of healthcare professionals and the importance of adequate communication with the patient*”. Overall, communication was an overwhelmingly common response from students as a takeaway from all simulation cases, both virtual and in-person, as well as outpatient and inpatient scenarios.

### 3.2. Subtheme: Self-Development

Self-development skills were a commonly mentioned topic for students in terms of expectations and self-reported outcomes for in-person and virtual simulation experiences. Many of the self-development skills that students referenced echoed the importance of more nuanced communication skills they hoped to take into their career fields.

In the virtual simulation settings, students expressed the desire for strategies and professional skills that can carry over to their future careers going into the simulation experience. Students desired “*information that will help me lead a team in implementing strategies/protocols that require multiple providers*”, while also hoping to experience their career-specific skills, such as being “*more comfortable as a Nurse Practitioner with diagnosing and treating*”. While these skills would directly benefit students’ own career skill development, students also mentioned wanting to learn and gain knowledge that would allow them to develop their skills to use in communicating with healthcare teams. One student wanted to “*gain better history-taking skills and use this as a learning opportunity*”. At the same time, another hoped to “*know a bit more about the healthcare operations in an ambulatory setting*”. The desire for self-development skills to increase personal knowledge and growth was an important aspect of what students expected from the virtual simulation experience.

Critical thinking skills were another aspect of self-development that students hoped to gain from the simulation experiences. This particular aspect of self-development skills only appeared in student responses to the in-person simulation experiences. Students expressed a desire to possess “*critical thinking skills and strength with collaborating with other members of the healthcare team*”, and, more specifically, a “*hope to utilize problem-solving and decision-making skills in regard to the simulated case*”. As students reported the outcomes after the simulations, critical thinking skills were reported by students as a specific experience gained, as well for both in-person and virtual simulations. Specifically, students only reported critical thinking skills as an outcome for the inpatient case scenario. Students mentioned being able to “*work up medical errors and figure out what could have gone wrong at many different levels*”, as well as learning to “*analyze, think critically, and plan as a health professional team*”. Across both expectations and self-reported outcomes, critical thinking was a vital component for students.

Leadership-specific self-development skills manifested in student responses as an expected tool to gain during the simulations for both virtual and in-person settings. In the inpatient case scenario, students expected to gain an “*increased ability to assign roles in a team setting*” and “*more leadership and teamwork experience*”. The desire for leadership skills is one area of self-development that students expected to gain from the inpatient simulation experience both virtually and in person.

Many centered their goals for the virtual simulation cases around providing better patient care to their future patients, another self-development skill. Students indicated that they hoped to gain insight into interacting “*with various healthcare workers in a professional manner while delivering high-quality care to patients*”, as well as being able to “*promote better communication between providers that ultimately results in improved patient care*”. Patient interactions were something that many students stated that they would like more experience with, including “*learning how to navigate patient encounters effectively*” and “*understanding how to treat patients with complicated medical history*”. In terms of self-reported outcomes, patient care was also identified in the outpatient case in both virtual and in-person settings. Students felt that “*it was a great learning experience on how to understand patients better and deal with their frustrations and concerns*”, as well as “*it’s important to listen to the patient. Obtain a full history and never ignore other health concerns*”. While it was frequent that the responses from students mentioned communication or teamwork, they were often focused on the end goal of delivering quality patient care via an interdisciplinary team.

Another skill under the self-development subtheme that emerged more frequently during the survey on what students expected to gain from the in-person simulation as opposed to the virtual simulation was the goal of being able to gain real-life experience and apply previously learned skills. Students mentioned wanting to “*practice implementing real-life nursing practices in a mock hospital setting*” and “*hoping to gain more clinical-setting experience*”. Ultimately, these students mentioned wanting to expand their “*experience, knowledge, and confidence*” as healthcare professionals.

An additional self-development skill that emerged as a self-reported outcome for students was centered around telehealth practices. This pattern only emerged as an outcome in the outpatient scenario and only in a virtual setting. After the virtual outpatient simulations, students recognized that “*telemedicine requires a different skill set to make sure the patient remains the center of care and feels heard and cared for. Furthermore, I learned that showing empathy digitally is different from showing empathy in person*”. Specifically, self-development skills and communication were aligned with what students learned about telehealth practices in that “*during the patient interaction itself, I learned how important it is to maintain eye contact in a virtual setting so that the patient still feels listened to and understood*”. The self-reported outcomes centered around telehealth practices for students were a unique pattern that aligned with virtual simulation implementation despite students not expecting to gain this skill ahead of time.

Recognizing the role of social determinants of health was a self-development skill that manifested in the outpatient scenario and only for in-person simulations. Students gained an understanding of “*the role of social determinants of health in our patient’s decision-making*”. Also, they learned “*how to better manage patients with social issues such as no insurance, older age with no close family members, etc.*”. As the outpatient scenario took place at a charity clinic and centered around a patient who had experienced job and insurance loss, students learning about social determinants of health was an important outcome.

Lastly, students appreciated the challenging nature of simulations, a self-development skill they can apply to their future careers. This self-reported outcome only occurred in the inpatient scenario and the virtual simulation setting. Students mentioned learning that “*it is a much more complicated task than I thought. It takes time to both reach an understanding as a group and also follow through with the activity at hand*” when referring to handling a medical error as a team. Additionally, one student echoed the complexity of the situation in that “*crisis management is very difficult, especially when health care providers make errors that need to be explained to patients*”.

### 3.3. Theme 2: Teamwork

Students acknowledged teamwork (n = 1072/1722; 62.3%) was a skill they hoped to gain from the experience. Across both in-person and virtual simulation formats, some students equated communication and teamwork as skills they hoped to simultaneously gain from the simulation experiences, as they hoped for “*improvement on communication, collaboration, team functioning, and conflict management*”, and many students exclusively emphasized their desire to gain skills specific to working with varied teams of healthcare professionals. One student participant noted the following:

As an aspiring physician, I believe hearing from those who are currently working in clinical settings will shape my understanding of healthcare delivery and patient care. Learning from them now will ultimately shape how I approach situations with patients in the future, so I am excited about that mentorship aspect as well.

After the simulation, when discussing the notion of teamwork (n = 959/1719; 55.8%), many students emphasized the importance of effective teamwork in providing effective patient care by stating, “*It is a good practice to work together, listen to each other, and achieve a common goal of patients getting better*”. These responses overwhelmingly support students’ desire to improve their communication and teamwork skills to provide optimal patient care.

Students acknowledged the benefit of learning to work effectively with a team after their simulation cases. One participant stated the following:

I learned that there will be situations that you will not know how to handle. Fortunately, there are always people that can help and guide you. I learned that working as a team definitely has its benefits. You will always have one person that will know enough to take care of any situation that arises. I learned that I should work with those around me and utilize the resources that are available to me so that I can provide the best care that I can.

The support a team can provide when handling difficult patient scenarios proved to be a valuable takeaway for participants after the simulation cases in that “*teamwork should be a priority*” when working with complicated patient cases.

### 3.4. Subtheme: Professionalism

Professionalism manifested in various ways, and, here, specifically, in terms of relating to working with an IPE team in handling patient frustration and anger and in student responses regarding expectations and outcomes in both in-person and virtual simulation settings.

One theme that emerged uniquely in the inpatient case simulation across both in-person and virtual settings was the concept of de-escalation of inpatient encounters. Students reiterated the importance of “*being calm and patient*” and “*taking the blame for things that go wrong on the side of the clinic/office*” as the best way to de-escalate situations where patient emotions may run high. Overall, students felt that this simulation helped to prepare them for a situation where calming down an angry patient is crucial to providing the medical care that they need:

I learned a lot about how to take responsibility for medical errors in a way that not only informs the patient of our mistake but also addresses all of their concerns. It was difficult to speak to an irate patient, but I feel that with this simulation, I learned how to handle the situation.

This skill is one that helps students understand that “*building a rapport with the patient when you approach them as a team is very important*”.

### 3.5. Theme 3: Role Identification

Two patterns regarding role identification emerged exclusively in the outpatient case on virtual and in-person platforms. One was the expectation that students would learn about the roles of different healthcare providers to work more effectively with them (n = 151/997; 15.1%). For example, one student mentioned the following:

I also am interested in learning how different roles are defined in different healthcare settings, such as an ambulatory one, and how the roles are adapted to enable comprehensive patient care. Lastly, I hope to better understand my team members’ clinical perspectives and how they impact patient care.

The outpatient case scenario responses were the only ones to indicate a desire to learn more specifically about the roles and responsibilities of other healthcare professionals to achieve the highest levels of patient care.

The second concept that emerged exclusively in the outpatient case simulation, in both in-person and virtual platforms, was the notion that students were able to learn about the roles of other healthcare professionals in their team (*n* = 201/999; 20.1%). Students had the opportunity to “*work with different specialties, especially ones that are not common in my everyday practice, such as dentistry*”. The different specialists that were incorporated into each interdisciplinary team allowed students to realize that “*everyone on the team can contribute their unique funds of knowledge, regardless of the trajectory of the situation*”. Ultimately, students were able to see the benefit of working with a varied team of providers:

It is great to have other experts on the team, such as social workers, dentists, and pharmacists to help counsel the patient simultaneously. This also emulated the medical home model, which I think is a more effective and time-efficient way of delivering holistic medical care to patients.

Students could see the benefits of teamwork incorporated into their interdisciplinary teams.

### 3.6. Subtheme: Professionalism

One theme that was unique to students’ responses after they had completed the case simulations was the importance of maintaining professionalism in their respective roles. These responses occurred across both in-person and virtual settings. Students mentioned that “*maintaining professionalism when a patient is exhibiting anger/frustration*” was crucial to working through a difficult case simulation. A large component of professionalism for students was being able to “*admit mistakes that the team made and work together with other team members to come up with a plan for the patient*”. The importance of taking responsibility and being professional emerged when students realized “*how easy it is to make a fatal mistake when there is a lack of communication*”. Overall, professionalism manifested in how students interacted and communicated with their interprofessional teams when handling complicated patient cases.

The “*importance of prep work*” was a big realization for students when working together during the virtual case simulation. This theme appeared exclusively during the virtual simulation, as opposed to no instances of this code occurring during the in-person simulation case. Students acknowledged that the team should “*come up with a plan so we can be organized with our responses to our patient*”. Ensuring that students took accountability for a mistake and “*analyzed its root causes with other members of the team*” helped them to “*develop measures to prevent this type of mistake in the future*”. Knowing what students should be responsible for in their specific roles aligns with what is expected of them as professionals.

The importance of having “*clear and accurate information*” is one that students also took away from the inpatient case simulation in both in-person and virtual settings. To truly meet the needs of the patients, healthcare professionals should “*know your lab values and medications, and question something when it is not right*”. Students felt that the Electronic Health Record (EHR) was an important skill to develop in order to provide effective patient care. They stated that “*Mistakes were made, and the ease of use of an EHR is really important*”.

Overall, communication, teamwork, and role identification were major themes that students not only expected from the simulations but also the ones they reported as outcomes. However, there were explicit differences in students’ expectations and self-reported outcomes between the inpatient and outpatient case scenarios. In the inpatient case scenario, students perceived that they had the opportunity to focus on developing leadership-specific skills and an understanding of the challenging nature of complex medical error cases, especially when handling these situations in a virtual environment. In turn, students also learned the importance of having clear and accurate information about a case, as well as de-escalating techniques. On the other hand, the outpatient case afforded students the opportunity to explore telehealth skills in a virtual setting and the social determinants of health in in-person situations. This scenario also allowed students to learn and explore different healthcare professional roles and how they can work collaboratively to provide excellent patient care.

## 4. Discussion

The research questions guiding this study are as follows: (1) What do students expect to learn when completing an in-person IPE activity compared to a virtual IPE activity?; and (2) How do the self-reported outcomes of students who completed an in-person IPE activity differ from those who completed a virtual IPE activity? This study examined the qualitative data regarding student expectations and self-reported outcomes after completing two case-based in-person and virtual IPE simulations. One case scenario focused on a medical error in an inpatient setting, and the other focused on an uninsured patient seen in an outpatient charity clinic. Common themes emerging from students’ expectations and perceptions of outcomes help shed light on issues and determine the impact of these simulations on learning. These findings add to the literature on the benefits of IPE learning activities in improving interprofessional communication and collaboration among healthcare students. They will also help determine future improvements in IPE when delivered in both in-person and virtual formats. Although several studies in the literature have assessed the differences in student expectations and perceptions between in-person and virtual simulations, very few have compared both the formats as well as two different case scenarios. Our study is unique in comparing both of these aspects in a large sample of interprofessional students.

Our findings found that some of the expectations of students prior to the simulation in both in-person and virtual formats were similar. Students expected to learn better communication and collaboration strategies from these simulations. However, some of the expectations differed in the two formats. The expectations of the students participating in the in-person simulations were focused on gaining “real-life experiences”. In contrast, the expectations of students participating in the virtual simulations were to learn more about “improved patient care”. These findings were similar to other studies comparing in-person and virtual simulations where students’ perceived outcomes of the benefits of engaging in both simulations included the value of communication and the importance of collaboration [17,18]. Students in these studies also perceived “hands-on experience with simulated patients” as important to their learning in the in-person format [18].

Student reflections after completing the simulation cases revealed that communication and teamwork were common learning outcomes in both virtual and in-person IPE sessions in outpatient and inpatient cases. These findings are similar to many other studies that have reported that students value the importance of collaboration and communication as important learning outcomes when participating in IPE activities [17,18]. These findings reinforce the need to include IPE in all healthcare education programs. Complex healthcare systems require collaboration among healthcare professionals to ensure patient safety and provide quality healthcare [19,20]. Constructivism learning theory explains that learning occurs through building on prior knowledge and from social interactions with others [2], whereas Kolb’s experiential learning theory explains that learners’ knowledge is created by the process of experience transformation consisting of four stages [3,4]. IPE activities prepare diverse healthcare professionals to collaborate with each other and these social interactions and experiences help them provide better quality of care to their patients and caregivers.

Role identification emerged as a theme in this study, where students expected to learn about the roles of other healthcare professionals in the team. Other studies have demonstrated that students value learning about and from other healthcare professions [17,18,21]. Interprofessional learning allows students to have an opportunity to learn about and develop respect for other professions. Interprofessional learning is a core competency identified by the Interprofessional Education Collaborative [22].

Telehealth adoption has seen tremendous growth since the COVID-19 pandemic. However, healthcare students have limited exposure to telehealth technologies prior to graduation. Students participating in the virtual simulations identified *telehealth* as a learning outcome. Students learned skills such as interacting with a patient via telehealth, making eye contact, and showing empathy. These reflections were echoed by students participating in other IPE activities [19,23]. Students perceived these telehealth-based IPE experiences to be helpful in preparing them for practice [19] and learning about real-life situations [23]. The virtual/telehealth delivery format of IPE will help learners prepare for telehealth practice and collaboration and help answer the call for increased training for healthcare professionals in telehealth delivery [23,24].

Self-development and professionalism skills were commonly mentioned topics by the students in both virtual and in-person simulations. Two subthemes that were unique to the inpatient medical error case scenario in both the virtual and in-person formats were *de-escalation* and *clear and accurate information.* In this scenario, the patient was angry, had significant body language indicating anger, and also was quite loud in expressing his anger at the students for causing harm to him. This required the students to de-escalate the situation, calm the patient, and perform a root-cause analysis. In the study conducted by Marshall et al., which assessed the impact of an IPE medical error simulation, students also reported learning important aspects of how to disclose an error honestly and being supportive to the patient and family to de-escalate the situation [25].

The self-development subtheme of *patient care* and the overarching theme of role identification were unique to the outpatient charity care case scenario in both virtual and in-person formats. In this scenario, the standardized patient was portrayed as an older woman with dental pain but had an emergency that was detected during the visit that needed immediate hospitalization. This required the interprofessional team to change plans quickly while reassuring the patient and prioritizing patient care. Learning about different roles has been a consistent learning outcome for students in various other studies evaluating IPE simulations [17,18,26]. In the study by Lee et al., students perceived that the IPE experience helped them provide safe, quality healthcare and improve patient care [26].

This study highlighted the explicit differences in students’ expectations and self-reported outcomes between the inpatient and outpatient case scenarios. The inpatient case scenario provided students with the opportunity to focus on developing leadership-specific skills, as well as leaving them with an understanding of the challenging nature of complex medical error cases, especially when handling these situations in a virtual environment. In turn, students also walked away with the importance of having clear and accurate information about a case, as well as de-escalating techniques. On the other hand, the outpatient case gave students the opportunity to explore telehealth skills in a virtual setting and the social determinants of health in in-person situations. This scenario also allowed students to learn and explore different healthcare professional roles and how they can work collaboratively to provide excellent patient care.

### 4.1. Contributions

A valuable finding from our study was that students learned skills such as interacting with a patient via telehealth, making eye contact, and showing empathy from the virtual sessions. The online/virtual format of IPE delivery will help to prepare students for telehealth practice and collaboration and also provide opportunities for distance education [22]. In addition to acquiring various self-development skills, this study also found that the students learned from the social interactions in both formats and were able to value the contributions of other health professionals in an interprofessional team, as well as realize the importance of their role in these teams.

### 4.2. Limitations

This study had a few limitations. The virtual and in-person IPE simulation delivery was not carried out in tandem or in the same year. One occurred during the pandemic when restrictions prevented in-person simulations and one where there were no in-person class restrictions. This could have affected the difference in findings. Data were not disaggregated by disciplines for this study. The results may not be generalizable to students with a different curriculum or with a different student population. Although similar training was provided to all the SPs, different SPs were involved in depicting the cases, which may be considered a limitation. Another limitation of this study is the reliance on self-reported data, which may introduce response bias and result in the potential variability in student experiences.

### 4.3. Recommendations for IPE

It is key for IPE activities to have clear goals and learning objectives. If the goal of the IPE activity is for students to have a telehealth experience with a learning objective, for example, to manage a patient encounter using telehealth technology, a virtual IPE activity may be the most appropriate modality. As one student expressed in the post-survey of a virtual experience, “*How to empathize with the patient over telemedicine–much harder than in person!*”. When transitioning from an in-person IPE simulation to a virtual format, IPE educators should employ strategies such as foreseeing potential challenges, staying committed to continuing IPE throughout challenges such as the pandemic, ensuring student support, using technology appropriately, and measuring outcomes [8]. Providing guidance, support, clear instructions, interaction, and supervision, and considering the student’s feedback [9], is very important in designing IPE activities. Collecting qualitative student feedback, as performed in this study, provides rich information for educators to help modify and redesign activities to achieve the learning objectives.

## 5. Conclusions

Our study found that students perceived teamwork, communication, and role identification as important learning outcomes when participating in IPE activities. Not only were results from in-person and virtual simulations supported, but these findings were consistent with the inclusion of two different case scenarios. Additionally, students’ expectations and perceived outcomes varied based on the case scenario that they experienced, allowing students exposure to real-life experiences and practices that help to improve patient care by practicing to the full extent of their education and training they may not have otherwise been exposed to. These experiences ultimately helped students to be more prepared to tackle complex healthcare situations with interprofessional teams, a crucial component in providing high-quality patient care. Realistic IPE simulations, in virtual or in-person formats, can thus contribute to students developing confidence in their role as an appreciated member of an interprofessional team, as well as learning about different roles. They also help teach communication and collaboration to provide excellent, safe patient care. Student expectations and their perceived outcomes from IPE activities are one component faculty and administrators should include for making well-informed curriculum decisions. Additionally, IPE simulations delivered virtually can develop students’ confidence in their telehealth and IPE knowledge and skills. The virtual setting can also provide an optimal venue for convening large groups while continuing to deliver authentic IPE. Future research should focus on the long-term outcomes of IPE activities on health profession students, the transfer of IPE and telehealth skills to individuals’ professional practice after graduation, and, ultimately, on improvements in patient care.

## Data Availability

Data are available upon request from the corresponding author.

## Supplementary Materials

The following supporting information can be downloaded at: https://www.mdpi.com/article/10.3390/nursrep15030114/s1, Supplementary S1: Pre-test and Post-test questions; Supplementary S2: Inpatient case scenario—Evans-Door note. Supplementary S3: Outpatient case scenario—Inez Peterson-Door note.

## Public Involvement Statement

There was no public involvement in any aspect of this research.

## Guidelines and Standards Statement

This manuscript was drafted in accordance with the Standards for Reporting Qualitative Research (SRQR) (O’Brien et al., 2014 [27]) for qualitative research.

## References

[1] Phanudulkitti C., Eze C.E., Farris K.B. (2023). Student Pharmacists’ Attitude Changes Toward Interprofessional Education Following an Introductory Interprofessional Course. Am. J. Pharm. Educ..

[2] Lebois L.A., Wilson-Mendenhall C.D., Simmons W.K., Barrett L.F., Barsalou L.W. (2020). Learning situated emotions. Neuropsychologia.

[3] Kolb D.A., Boyatzis R.E., Mainemelis C. (2014). Experiential Learning Theory: Previous Research and New Directions.

[4] Dong H., Lio J., Sherer R., Jiang I. (2021). Some Learning Theories for Medical Educators. Med. Sci. Educ..

[5] Gilbert J.H.V., Yan J., Hoffman S.J. (2010). A WHO Report: Framework for Action on Interprofessional Education and Collaborative Practice. J. Allied Health.

[6] Marion A.D.C., Pereira L.C., Pinho D.L.M. (2023). The effect of interprofessional simulation practice on collaborative learning: A randomized controlled trial. J. Interprofessional Care.

[7] Abdelaziz A., Mansour T., Alkhadragy R., Nasser A.A., Hasnain M. (2021). Challenges to Interprofessional Education: Will e-Learning be the Magical Stick?. Adv. Med. Educ. Pr..

[8] McKinlay E., Banks D., Coleman K., Darlow B., Dungey G., Farr T., Fyfe R., Gray B., Kemp L., Mitchell M. (2021). Keeping it going: The importance of delivering interprofessional education during the COVID-19 pandemic. J. Prim. Health Care.

[9] Power A., Park V., Owens M., Sy M.P. (2022). Academics’ experiences of online interprofessional education in response to COVID-19. Br. J. Midwifery.

[10] Wetzlmair L.-C., Kitema G.F., O’Carroll V., El-Awaisi A., Power A., Owens M., Park V., McKinley M., Anderson E.S., Loder-Fink B. (2021). The impact of COVID-19 on the delivery of interprofessional education: It’s not all bad news. Br. J. Midwifery.

[11] Champagne-Langabeer T., Neher S.E., Cardenas-Turanzas M., Swails J.L. (2022). Unintended Consequences of a Transition to Synchronous, Virtual Simulations for Interprofessional Learners. Healthcare.

[12] Menon R., Emerson G., Yee J. (2023). Ventricular Tachycardia. J. Educ. Teach. Emerg. Med..

[13] Champagne-Langabeer T., Revere L., Tankimovich M., Yu E., Spears R., Swails J.L. (2019). Integrating Diverse Disciplines to Enhance Interprofessional Competency in Healthcare Delivery. Healthcare.

[14] Tisdell E.J. (2016). Qualitative Research: A Guide to Design and Implementation.

[15] Munro M., Cook A.M., Bogart K.R. (2022). An inductive qualitative content analysis of stigma experienced by people with rare diseases. Psychol. Health.

[16] Lungu M. (2022). The Coding Manual for Qualitative Researchers. Am. J. Qual. Res..

[17] Andreeff R., Edwards S. (2023). Student Perceptions of Online (Virtual) vs. In-Person Interprofessional Education Simulation During the COVID-19 Pandemic. J. Allied Health.

[18] Hughesdon K., Zakrajsek A., Washington V.L., Seurynck K., Myler L., Holt S. (2023). Interprofessional simulation with nursing and occupational therapy students: Comparing a virtual and in-person event. J. Interprofessional Educ. Pr..

[19] Scott A., Dawson R.M., Mitchell S., Catledge C. (2020). Simulation-Based Interprofessional Education in a Rural Setting: The Development and Evaluation of a “Remote-In” Telehealth Scenario. Nurs. Educ. Perspect..

[20] Kaldheim H.K.A., Fossum M., Munday J., Johnsen K.M.F., Slettebø Å. (2021). A qualitative study of perioperative nursing students’ experiences of interprofessional simulation-based learning. J. Clin. Nurs..

[21] Morrell B.L.M., Nichols A.M., Voll C.A., Hetzler K.E., Toon J., Moore E.S., Moore S.M., Kemery S.R., Carmack J.N. (2018). Care Across Campus: Athletic Training, Nursing, and Occupational Therapy Student Experiences in an Interprofessional Simulation. Athl. Train. Educ. J..

[22] Collaborative I.E. IPEC Core Competencies for Interprofessional Collaborative Practice: Version 3. Interprofessional Education Collaborative. https://www.ipecollaborative.org/ipec-core-competencies.

[23] Wong L., Tokumaru S., Boehm L., Young N., Todoki S., Meguro A., Thai L., Loos J.R., Masaki K. (2021). From a distance: Nursing and pharmacy students use teamwork and telehealth technology to provide interprofessional care in a simulation with telepresence robots. J. Interprofessional Educ. Pr..

[24] Hyde M.J., Cook K., Dickey P., Fingeret A., Reynolds J., Bronner L.P. (2023). Interprofessional telehealth simulation for students focusing on Progression of care from a rural primary care clinic to an urban hospital. J. Interprofessional Educ. Pr..

[25] Marshall C., Van Der Volgen J., Lombardo N., Hamasu C., Cardell E., Blumenthal D.K. (2020). A Mixed Methods Approach to Assess the Impact of an Interprofessional Education Medical Error Simulation. Am. J. Pharm. Educ..

[26] Lee W., Kim M., Kang Y., Lee Y.-J., Kim S.M., Lee J., Hyun S.-J., Yu J., Park Y.-S. (2020). Nursing and medical students’ perceptions of an interprofessional simulation-based education: A qualitative descriptive study. Korean J. Med. Educ..

[27] O’Brien B.C., Harris I.B., Beckman T.J., Reed D.A., Cook D.A. (2014). Standards for Reporting Qualitative Research: A Synthesis of Recommendations. Acad. Med..

