# Supplementary material for: Student Expectations and Outcomes in Virtual vs. In-Person Interprofessional Simulations: A Qualitative Analysis
Source: Nurs Rep. 2025 Mar 20;15(3):114. doi: 10.3390/nursrep15030114 (PMC11944972; doi:10.3390/nursrep15030114)
Supplement: Supplementary file 1 [file nursrep-15-00114-s001.zip › Supplementary S1 - Pre-test and Post-test questions.pdf]

Supplementary S1

Inpatient medical error case scenario (M. Evans)
Outpatient charity care case scenario (I. Peterson)

Pre-Test Questions:

1. Please select the UTHealth School(s) that you are currently attending.

2. If you are a dual enrollment student, please select the second UTHealth School that you are enrolled in

3. Age
Please write your answer in whole numbers.

4. What is your gender?

5. Have you previously participated in any interprofessional trainings or activities through UTHealth or other format? (i.e. previous IPE simulations, IPE formal training, IPE lectures/ exercises).

6. What are you hoping to gain from this experience?

7. Based on the learning objectives below, which of the following skills are the most important to you? Please choose **at least** 3.

1. Communication
Ability to communicate effectively in a respectful and responsive manner with others ("others" includes team members, patient/client, and health providers outside the team).

2. Collaboration
Ability to establish/maintain collaborative working relationships with other providers, patients/clients and families.

3. Roles and Responsibility
Ability to explain one's own roles and responsibilities related to patient/client and family care and to demonstrate an understanding of the roles, responsibilities and relationships of others within the team.

4. Collaborative Patient/Client-Family Centered Approach
Ability to apply patient/client-centered principles through interprofessional collaboration.

5. Team Functioning
Ability to contribute to effective team functioning to improve collaboration and quality of care.

8. If you chose "Other" previously, please explain what skill(s) is most important to you:

Inpatient medical error case scenario (M. Evans)
Outpatient charity care case scenario (I. Peterson)

Post-test Questions

1. What did you learn from this IPE activity that you can apply to your professional career?

2. Based on the learning objectives below, which areas do you feel your interprofessional education (IPE) skills were enhanced or improved? Please select all that apply.
   Communication
   Collaboration
   Roles and Responsibility
   Collaborative Patient/Client-Family-Centered Approach
   Team Functioning
   Conflict Management and Resolution
   Other

3. If you chose “other” previously, please explain what skill(s) was improved or enhanced in this IPE activity

4. Please give at least one potential area for improvement for this activity

5. Did you play a role other than your role as a student (e.g., I am a nursing student playing the role of pharmacist)? Yes/No

6. If you answered “yes” to question above, which role did you play and how did that affect your experience in the simulation today?

7. Overall, please rate the effectiveness of your team.
   Excellent
   Very Good
   Good
   Fair
   Poor
8. Please explain your team rating in a few sentences
