# Supplementary material for: Student Expectations and Outcomes in Virtual vs. In-Person Interprofessional Simulations: A Qualitative Analysis
Source: Nurs Rep. 2025 Mar 20;15(3):114. doi: 10.3390/nursrep15030114 (PMC11944972; doi:10.3390/nursrep15030114)
Supplement: Supplementary file 1 [file nursrep-15-00114-s001.zip › Supplementary S2.pdf]

20-21 IPE INPATIENT, M. Evans – Door Note

PATIENT INFORMATION

Name: M. Evans

Setting: Hermann Hospital Patient Room, accessed via telemedicine due to potential COVID exposure

Time: During regular daytime hours

CHIEF COMPLAINT

Mr. Evans is a 61 year old male who was admitted to the hospital with endocarditis, planning to be discharged today. He was at an international conference the day before admission, and remains on precautions for COVID-19 pending test results.

VITAL SIGNS (Patient took her own):

BP: 126/86

Pulse: 70 and regular

Resp: 18

Temp: 98.0 degrees F

Student INSTRUCTIONS

Tasks:

1. Introduce yourself and your role via the chat box
2. Inform the patient of the error made in medication dose during this hospitalization, and that he must stay overnight for observation

TIME LIMIT: 15 minutes

PATIENT FEEDBACK: 15 minutes
