# Supplementary material for: Student Expectations and Outcomes in Virtual vs. In-Person Interprofessional Simulations: A Qualitative Analysis
Source: Nurs Rep. 2025 Mar 20;15(3):114. doi: 10.3390/nursrep15030114 (PMC11944972; doi:10.3390/nursrep15030114)
Supplement: Supplementary file 1 [file nursrep-15-00114-s001.zip › Supplementary S3.pdf]

20-21 IPE OUTPATIENT, I. Peterson – Door Note

PATIENT INFORMATION

Name: I. Peterson

Setting: Student-Run Free Clinic, operating via telemedicine – your attending obtained consent and ensured patient privacy

Time: During regular daytime hours

CHIEF COMPLAINT

Mrs. Peterson is a 71-year-old female who comes to the clinic to establish care for osteoporosis and tooth pain.

VITAL SIGNS (Patient took her own):

BP: 126/86

Pulse: 70 and regular

Temp: 98.0 degrees F

Student INSTRUCTIONS

Tasks:

1. Introduce yourself and your role via the chat box
2. Take a relevant history
3. DO NOT PERFORM a physical examination
4. Discuss with patient possible diagnosis and treatment plan

TIME LIMIT:

First 5 minutes: Dentist to take History

Next 15 minutes: Other students will join the dentist to discuss History and come up with an assessment and plan.

PATIENT FEEDBACK: 10 minutes
